# Selective Sweep in the *Flotillin-2* Region of European *Drosophila melanogaster*

**DOI:** 10.1371/journal.pone.0056629

**Published:** 2013-02-21

**Authors:** Annegret Werzner, Pavlos Pavlidis, Lino Ometto, Wolfgang Stephan, Stefan Laurent

**Affiliations:** Section of Evolutionary Biology, Department of Biology II, University of Munich, Planegg-Martinsried, Germany; North Carolina State University, United States of America

## Abstract

Localizing genes that are subject to recent positive selection is a major goal of evolutionary biology. In the model organism *Drosophila melanogaster* many attempts have been made in recent years to identify such genes by conducting so-called genome scans of selection. These analyses consisted in typing a large number of genetic markers along the genomes of a sample of individuals and then identifying those loci that harbor patterns of genetic variation, which are compatible with the ones generated by a selective sweep. In this study we conduct an in-depth analysis of a genomic region located on the X chromosome of *D. melanogaster* that was identified as a potential target of recent positive selection by a previous genome scan of selection. To this end we re-sequenced 20 kilobases around the *Flotillin-2* gene (*Flo-2*) and conducted a detailed analysis of the allele frequencies and linkage disequilibria observed in this new dataset. The results of this analysis reveal eight genetic novelties that are specific to temperate populations of *D. melanogaster* and that may have arisen during the expansion of the species outside its ancestral sub-Saharan habitat since about 16,000 years ago.

## Introduction

Localizing genes that are subject to recent positive selection is a major goal of evolutionary biology. Recent methodological and theoretical advances are facilitating their identification by allowing the shift from candidate-locus approaches to genome-wide analyses. This is particular relevant for studying the effects of recent positive selection, which may be hampered by the confounding effects of demography. Within a population an ecologically favored allele might rise in frequency and become fixed in the population. This process is often caused by environmental changes or colonization of new environments. In case the favored allele already exists in the population selection operates on standing genetic variation, otherwise a favored mutation must arise *de novo*. If selection is strong, the fixation of the beneficial mutation can have a joint effect on linked neutral sites, which are also expected to increase in frequency. This process, known as genetic hitchhiking [1], generates a signal of i) reduced genetic variation at the target of selection [2], [3], ii) a skewed site frequency spectrum (SFS) due to an excess of rare and high-frequency derived alleles [4], [5], [6], and iii) increased linkage disequilibrium (LD) on both sides of the target of selection but reduced LD between them [7], [8], [9].

The search for adaptive signals is usually carried out using a large number of loci on a genome-wide scale to identify regions that show considerable deviation from neutral expectations. As generally known, however, standard neutrality defined by the Wright-Fisher model is rarely met in natural populations, thus cautioning on the use of the classical neutrality tests. In fact, population structure and demographic events such as population bottlenecks or population size expansion can mimic genetic footprints of selection and possibly lead to false positives [5], [10], [11]. However, a useful neutral expectation may be provided by a demographic null model that is fitted to the demographic history of a population [12].

This approach has been used by Glinka *et al.* [13] and Ometto *et al.* [14] who performed a chromosome-wide scan of DNA variation in a derived European population of *Drosophila melanogaster* and compared it to a putative ancestral population from Southeast Africa. More than 250 loci on the X chromosome were used to evaluate the contribution of adaptive evolution in the European population. The demographic null model of the European population was defined as a bottleneck caused by the post-glacial colonization of Eurasia around 10,000–15,000 years ago [14], [15], [16], [17]. While the bottleneck could account for most of the reduction of variation observed in the European population, the analysis revealed several loci and regions whose very low level of genetic variation and highly skewed SFS were not compatible with the expectations under such a demographic scenario.

In this follow-up study, we concentrate on one of these low-variation regions comprising about 20 kb centered around the cytological position 13A1 between landmarks X:14810552 and X:14829908 (FlyBase, FB2012_06, released November 6th, 2012). Overall, five genes are located within this region: *CG9504, CG9503, CG32591, CG9009,* and *CG32593*.

*CG9504* and *CG9503* are two of the 12 genes that were assigned to the GMC oxidoreductase family and are clustered on the X chromosome of *D. melanogaster* [18]. The functions of these GMC homologues are still largely unknown except for *CG9504*, which was described as ecdyson oxidase (Eo) [19]. An expression analysis showed an up-regulation of both *Eo* and *CG9503* during embryonic and metamorphic development, possibly indicating a joint function in the ecdysteroid metabolism [18]. Remarkably, the GMC cluster is located within the second intron of the *Flo-2* gene with opposing transcription orientations. This arrangement is conserved throughout four distantly related insect species: *D. melanogaster*, *Anopheles gambiae*, *Apis mellifera*, and *Tribolium castaneum* [18], consistent with the occurrence of strong purifying natural selection.

*CG9009*, named *pudgy* (*pdgy*), encodes a long chain fatty acid CoA ligase and is up-regulated in the case of food deprivation [20]. As transcriptional target gene of the insulin pathway, the expression of *pdgy* is crucial for several metabolic parameters, such as glycogen storage and lipid homeostasis [21]. In general, nutritionally regulated genes are expected to orchestrate the energy homeostasis control, including the ability to mobilize stored energy resources, and *pdgy* is of vital importance in this process.

*CG32593, flotillin-2* (*Flo-2*), is highly conserved among a wide range of species (orthologous to the vertebrate *reggie 1*) and encodes for Flotillin-2, a scaffolding protein that is involved in the formation of non-caveolar lipid rafts [22]. In *Drosophila*, *Flo-2* has been found to be required to delimit the spread of epidermal wound response [23] and is an important component of the morphogens Wnt and Hedgehog [24]. It is a single transmembrane protein that is characterized by a short membrane-anchoring segment at the N-terminal part of the protein and a large cytoplasmic C-terminal domain [25]. The exact mode of membrane association is dependent on co-translational protein modifications at highly conserved N-terminal regions. If these regions are altered by mutations or if the protein is truncated the ability of membrane anchoring is lost. *Flo-2* is expressed predominantly in neuronal structures such as the optic lobes and the central brain during all developmental stages of *D. melanogaster* [26]. Overexpression of *Flo-2* leads to detrimental effects during the development of eyes, ocelli, bristles, and wings [27], while knockout mutants surprisingly show no noticeable phenotypic abnormalities. As *Flo-2* covers almost the entire genomic section of the candidate region we will refer to it as the ‘*Flo-2* region’.

Using the full-length sequence of the *Flo-2* region in both the European and African population samples mentioned above we applied two statistical tests for selection: 1) SweepFinder based on information of the SFS [28], and 2) the ω statistic based on measures of LD [7], [29]. The demographic null-model was inferred by simulations from the colonization model suggested by Laurent *et al.* [30]. Similar approaches were successfully used in other case studies of selective sweeps in *D. melanogaster* in our laboratory [31], [32], [33].

## Materials and Methods

### Fly Strains and Conditions of Culture

Intraspecific data were collected from highly inbred *D. melanogaster* lines, 12 derived from a European population (Leiden, The Netherlands) and 12 from an African population (Lake Kariba, Zimbabwe). All stocks were kept at 23°C, 45% humidity, and under constant light conditions. Development took place on a high-nutrient killed yeast food medium (12 ml) in glass vials of 200 ml. For interspecific comparison, we used the annotated sequence of *D. sechellia* (http://flybase.org, Release 5.31) [34].

### Sequence Data Collection and Analysis

Primers were designed based on the *D. melanogaster* genome (FlyBase, Release 5.1). We amplified and sequenced the complete genomic region X:14810552-X:14829908 using 36 partially overlapping DNA fragments (primers are given in Table S1). DNA sequences were obtained from individual male flies and generated as described in Glinka *et al.* [13]. Sequences were assembled into contigs using the program Seqman (DNAstar, Madison, WI, USA). Finally, sequences for all lines were aligned using the algorithm MUSCLE (Edgar 2004) with the online tool available at http://mobyle.pasteur.fr/cgi-bin/portal.py [35], creating a 20,011 bp alignment of 24 *D. melanogaster* lines and *D. sechellia* as outgroup. Sequences have been deposited in GenBank (accession numbers: KC460991-KC461010).

Basic population genetic parameters were estimated by a sliding window analysis (window size of 1,000 bp with 500 bp overlap) using the program DnaSP 5.0 [36]. We estimated nucleotide diversity using π [37] and θ_W_ [38]. The allele frequency distribution was measured with the summary statistic Tajima’s *D* [39] and based on the total number of segregating sites. The interspecific divergence to *D. sechellia* was determined for all 24 inbred lines of *D. melanogaster*. Furthermore, we used *D. sechellia* to polarize the state of the segregating sites in our population sample. A variant was considered ancestral if it was shared between both species and derived if it was present only in *D. melanogaster*.

A Wilcoxon rank sum test was performed to compare the nucleotide diversity of the *Flo-2* region with the entire X chromosome [14]. To avoid multiple testing due to window overlaps, only non-overlapping neighboring windows were selected for the analysis.

### Demographic Modeling

The selective sweep detection methods used in this study (*i.e.* SweepFinder and ω) requires the specification of a neutral demographic model. For this we used a slightly modified version of the demographic model of Laurent *et al.* [30], which describes our current understanding of the demographic history of African, European, and Asian natural populations of *D. melanogaster*. In this study we re-estimated the parameters of the Laurent *et al.* [30] model using an Approximate Bayesian Computation (ABC) approach [40], while adding to the summarized dataset the number of fragments that are monomorphic in the African, European, and Asian samples. These fragments are generally removed from ABC estimations because standard statistics like Tajima’s *D* [39] are undefined when the number of segregating sites is zero, which in turn causes technical problems in subsequent calculations. Nevertheless, the frequency of such monomorphic fragments within a genome scan does carry information about the past action of genetic drift within a population and should be taken into account in ABC model inference. This inclusion forces our demographic model to account for the fact that genomic regions lacking genetic variation, like the *Flo-2* region, can arise due to genetic drift alone without invoking the action of positive selection. Times of divergence and effective population sizes of the three populations were estimated applying the ABC algorithm described in Laurent *et al.* [30] to a genome-wide nucleotide polymorphism dataset taken from previous studies [13], [14], [30], [41]. The observed values for the number of monomorphic fragments within the African, European, and Asian populations are 0, 16, and 46, respectively. The prior distributions used for the ABC estimations are described in Table S2.

### Selective Sweep Analysis

First, the European dataset was analyzed using the SweepFinder algorithm [28]. Typically, SweepFinder compares the SFS of a small region of the genome (‘window’) to the SFS of the rest of the chromosome, which is considered neutral. In this study, the neutral SFS has been estimated using simulations of the above-mentioned version of our demographic model for the European population. For data analysis, we defined 1,000 overlapping windows of variable sizes according to the strength of selection and recombination rate.

We calculated the composite likelihood ratio (CLR) for each of these 1,000 windows along our sequence alignment for two models: a model without selection based on the neutral SFS *vs*. a model of a recent selective sweep, as suggested by Kim and Stephan [6] and later modified in SweepFinder by Nielsen *et al*. [28]. Thus, we consider the spatial pattern of allele frequencies along the studied genomic sequence, as predicted by a selective sweep model given the background pattern of a bottleneck. Incorporating the demographic history of the European population in the estimation of the null distribution controls the false-positive rate [29]. As the European sample lacks genetic variation for a large part of the sequence we included monomorphic sites in our analysis. We used the combined dataset of the European and African samples to determine the state of monomorphic European sites that were polymorphic in Africa. This is expected to increase the power for detecting the signature of a selective sweep.

Second, the European dataset was subjected to an analysis of LD using the ω statistic developed by Kim and Nielsen [7]. The selective sweep model predicts elevated levels of LD within the two flanking regions of the selected site, while LD is not expected to extend across the two regions. This pattern is identified based on high values of ω. As before, the dataset was split into 1,000 overlapping windows spanning between 2,000 and 10,000 bp. The borders of the two flanking regions were allowed to vary, and the window size was chosen according to the size for which ω assumes the maximum value [29].

Statistical significance of the maximum values assumed by both statistics, CLR_max_ and ω_max_, was inferred from 10,000 neutral coalescent simulations using a slightly modified version of the demographic model of Laurent *et al*. [30]. To do these simulations we fixed θ using the effective population sizes estimated/used by the Laurent *et al.* model and divergence-based mutation rate estimates. This approach was preferred over the one that fixed the number of segregating sites, which has been shown to be associated with statistical biases [42]. The recombination rate (3.64×10^−8^) was obtained from the *D. melanogaster* recombination rate calculator [43]. Only the European subset of each simulation was used to assess the 95th percentile of the null distribution.

## Results

### Sequence Data Collection and Analysis of the *Flo-2* Region

**Figure 1:**
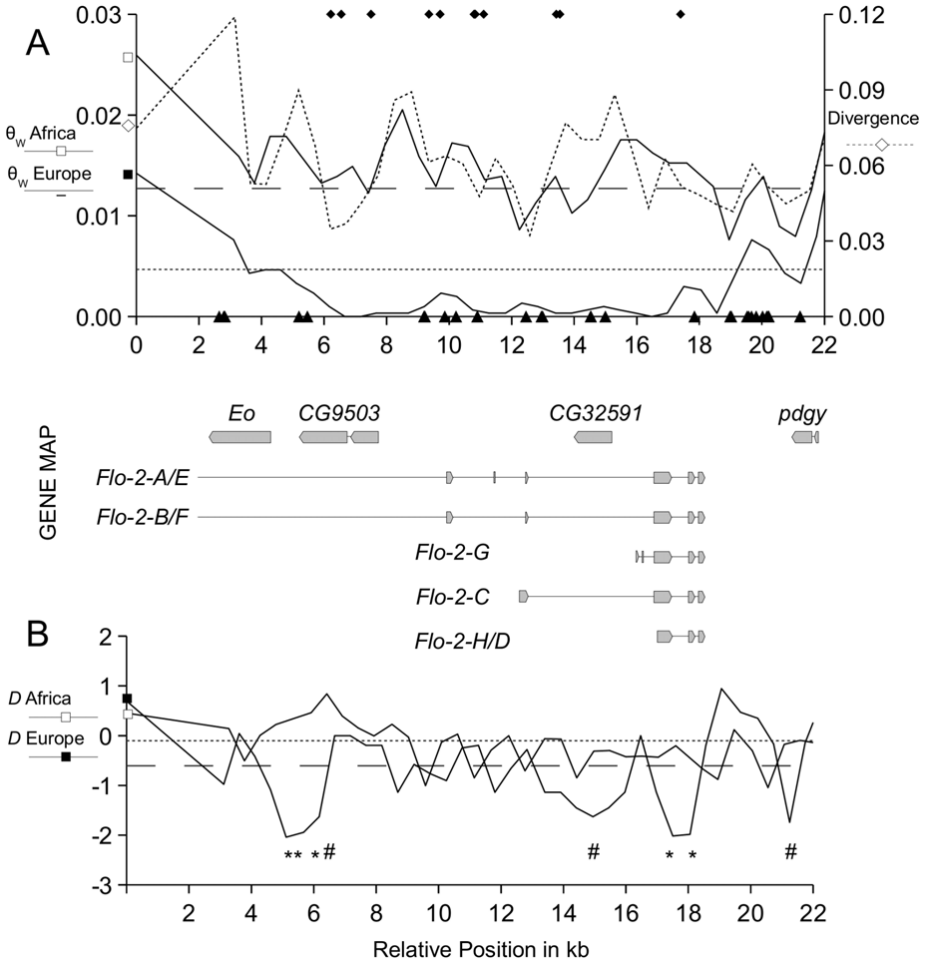
Nucleotide variation of the *Flo-2* region in an African and European sample of *D. melanogaster.* A) The valley of reduced genetic variation in a European sample of *D. melanogaster* compared to an ancestral population sample from Africa (solid lines; first y-axis, θ*_W_*). The fully sequenced 20,011-bp fragment of the *Flo-2* region is connected with an adjacent fragment of Ometto *et al.* [14], indicated by squares at coordinate 0 (white square-Africa; black square-Europe, distance to the fully sequenced *Flo-2* region around 3,000 bp, θ_W_ values were interpolated between the two fragments). The x-axis shows the relative position on the X-chromosome. The dashed horizontal lines show the average nucleotide variation of the X-chromosome for the African population sample (wide dashed line, θ_AX_ = 0.0131) and the European population sample (dashed line, θ_EX_ = 0.0047). Black triangles show the position of indels; black diamonds indicate fixed nucleotide differences between both samples. The second y-axis corresponds to divergence of *D. melanogaster* to *D. sechellia* (dashed line). The gene map gives the name and the position of the coding sequence of all genes located within the valley of reduced variation. These genes are *Eo*, *CG9503 CG32591*, *pdgy* (partly) and eight different transcripts of *Flo-2*. B) Tajima’s *D* statistic (solid lines) is shown for the African and European sample of *D. melanogaster* (white square-Africa; black square-Europe). The horizontal lines show the average value of Tajima’s *D* for the X chromosome of the African population sample (wide dashed line, *D*_XA_ = −0.608) and the European population sample (dashed line, *D*_XE_ = −0.103). Significance values are indicated as follows: # *P*<0.10; *P<0.05; **P<0.01.

To detect positive selection in the European sample of *D. melanogaster* we sequenced one of the low-variation regions discovered by Ometto *et al.* [14] using 12 inbred lines from the Netherlands (Leiden) and 12 inbred lines from the ancestral African range (Zimbabwe). The full sequence comprises 20,011 bp in total and is referred to as the *Flo-2* region. It is closely located to one of the fragments sequenced by Ometto *et al.* [14] that was added to Figure 1.

The sliding window analysis showed a conspicuous reduction of genetic variability for the European sample compared to the African one, with a valley of low variation of around 10 kb in size (6.7 kb–16.8 kb; see line with black square, Figure 1). In the European sample the mean value of nucleotide variation in the *Flo-2* region is θ_E_ = 0.0024, which is significantly lower than the observed value for the entire X chromosome in European *D. melanogaster* (θ_EX_ = 0.0047, [14]; fine dashed horizontal line in Figure 1; Wilcoxon rank sum test, *P* = 0.0026). The corresponding genetic region in the African sample has a nucleotide diversity of θ_A_ = 0.0141, which matches the mean value of the entire X chromosome (θ_AX_ = 0.0131 [14]; wide dashed horizontal line, Figure 1; Wilcoxon rank sum test, *P* = 0.1766). Our fully sequenced fragment covers several genes including *Eo*, *CG9503*, *CG32591*, parts of *pdgy* and eight different transcripts of *Flo-2* (Figure 1, Gene map, from left to right). Nonetheless, functional constraint as the sole cause of this pattern of genetic variability in the European sample can be ruled out as the level of genetic variation of the African sample is intermediate to high, and divergence to the sibling species *D. sechellia* remains constantly high at around 6%.

Tajima’s *D* statistic is negative in the European sample for most parts of the *Flo-2* region with an average value of *D*_E_ = −0.6416 (*D*_ XE_ = −0.103, [14]; Figure 1, *D* Europe). The African sample shows an average value of *D*_A_ = −0.2088 in the *Flo-2* region, a value that is above the European value and the African chromosomal average (*D*_ XA_ = −0.608, a value that was considered to reflect population growth [14]; Figure 1, *D* Africa). Thus, in the *Flo-2* region hallmarks of positive directional selection (including reduced variation and skews in the SFS) are only observed in the European population.

### Demographic and Selective Sweep Analyses

**Figure 2:**
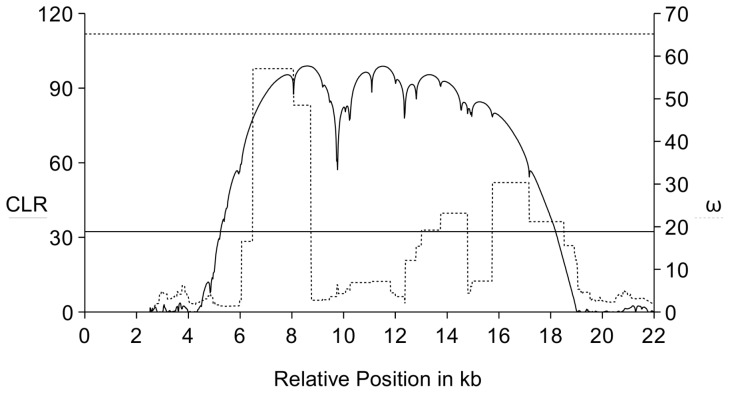
The sweep analysis reveals two profiles: the solid line shows the CLR of SweepFinder, the dashed line shows the result of the *ω* statistic. The horizontal lines give the 5% significance threshold for the CLR test (solid line, TH_CLR_ = 32.3) and the *ω* statistic (dashed line, TH_ω_ = 65.2).

The results of our demographic analysis are summarized in Table 1. Adding the number of monomorphic fragments to the vector of observed statistics in our ABC inference procedure did not change much the demographic estimates proposed by Laurent *et al.* [30]. The SweepFinder statistic for the *Flo-2* region is significant at the 5% threshold obtained by neutral simulations (see Materials and Methods; Figure 2, CLR). The ω statistic assumes relatively high values, but is not significant (Figure 2).

**Table 1:** Results of the demographic analysis.

	Chromosome X			Chromosome 3		
Parameters	mode	Q 2.5%	Q 97.5%	mode	Q 2.5%	Q 97.5%
Current African population size	4,635,114	2,392,436	28,700,928	4,346,139	2,141,666	28,089,983
Current European population size	1,586,481	760,318	4,866,692	1,200,356	577,222	4,831,432
Current Asian population size	338,810	91,417	4,540,468	530,964	90,852	4,745,731
Bottleneck size of the European population	22,975	10,898	87,811	38,948	21,591	95,993
Bottleneck size of the Asian population	10,741	3,554	90,142	13,887	5,207	91,777
Exit out of Africa	15,628	7,700	36,616	13,894	7,359	24,953
Colonization time of the South-Asian continent	3,264	1,194	7,518	4,188	1,135	8,594
Time of the expansion of the African population	27,643	2,383	369,569	33,536	3,869	378,727
Size of the ancestral African population	1,890,641	580,795	2,457,912	2,272,767	1,013,009	3,477,120

The parameters of the neutral demographic model that was used for the sweep analysis are given for the African, European and Asian population of *D. melanogaster*. The exit out of Africa, the times of colonization (times of divergence) and the respective population sizes were estimated by means of ABC using data from the X chromosome and chromosome 3.

NOTE.–The time estimations (*i.e*., modes and credibility intervals) are provided in years assuming ten generations per year. Population sizes are given in effective numbers of individuals.

### Private European Alleles

Geographically restricted genetic hitchhiking suggests that the target of selection should be fixed in the European population sample and absent in the African population sample. Overall there are 11 fixed different nucleotide substitutions between the African and the European sample within the *Flo-2* region (1–11, Table 2; black diamonds in Figure 1). Four of them are located within exons, while the remaining are located within *Flo-2* introns. In eight such cases the European population differs from the ancestral state of the African population (*i.e*., African *D. melanogaster* and *D. sechellia* share the same state), suggesting that they are derived private European alleles. The overrepresentation of eight derived alleles in the 10-kb segment of reduced variation within the20-kb long *Flo-2* region is highly significant (binomial distribution, *P* = 0.004). In the following we describe these fixed differences in detail according to their location across the genes present in the *Flo-2* region.

**Table 2:** Fixed differences between European and African.

nr.	abs. position	rel. position	location	Outgroup	Africa	Europe	consequence
1	14814106	6218	*CG9503*	G	G	A	Ser → Asn
2	14814438	6550	*CG9503*	T	C	T	syn. (Phe)
3	14815391	7504	*CG9503*	C	T	C	syn. (Asn)
4	14817203	9356	Intron	G	G	A	
5	14817533	9713	Intron	A	A	G	
6	14818608	10798	Intron	A	A	C	
7	14818649	10839	Intron	G	G	T	
8	14818898	11107	Intron	G	G	A	
9	14821087	13429	Intron	C	T	C	
10	14821196	13539	Intron	C	C	T	
11	14824970	17399	*Flo-2*	C	C	A	syn. (Ala)
12	14825835	12986	*Flo-2*	–	–	Ins.	Additional AS: THTHTNT

The derived state is highlighted in grey.

#### CG9503

Three fixed differences are located within the gene *CG950*3: one within the first exon, and two within the second exon. Within the second exon the substitution that is private to the European sample leads to an alteration of the amino acid sequence. Namely, a serine (Ser = ancestral state) is substituted by an asparagin (Asn) at the amino acid position 473. This substitution is not expected to alter the gene function as both amino acids have equivalent characteristics (hydrophilic, aliphatic, polar, neutral). The two other fixed differences are synonymous substitutions in the African sample while the European alleles are of ancestral state.

#### Flotillin-2

The intronic region of *Flo-2* carries seven fixed differences between the European and the African populations, six of which are of derived state in the former (Table 2). One fixed difference is located at a synonymous site of *Flo-2* without causing an amino acid substitution. Another fixed difference between the European and African samples is an indel polymorphism due to a dinucleotide microsatellite within the first exon of the transcript C of the *Flo-2* gene (Figure 1, gene map, *Flo-2-C*), and which results in the exon being 20 bp longer in the European sample than the African sample. Since the African *D. melanogaster* lines share the same state with *D. sechellia*, the most parsimonious hypothesis is that European lines gained the additional nucleotides. Interestingly there are a total of 38 indels spread along the *Flo-2* region (Figure 1, black triangles on the x-axis) but only this one is fixed between the African and European samples (Table 2, nr. 12). The annotated version of transcript form *Flo-2-C* at Flybase (version FB2010_05, released May 28th, 2010) is strongly supported by a full-length cDNA clone generated and sequenced by Rubin *et al.* [49]. The sequence has been subjected to integrity checks for accuracy, the presence of an open reading frame (ORF) as well as the start and a stop codon. Interestingly, not all of our studied *D. melanogaster* lines have an ORF (Table S3, *Flo-2-C* alignment starts at transcription site +46). Specifically, seven African lines show premature termination codons and two lines have frame shifts due to microsatellite length polymorphism leading to a stop codon loss. The remaining three African lines have a gene version that forms the *Flo-2-C* splicing variant in the correct way (lines 186, 377, 384). In contrast, in the European sample the majority of the lines – ten out of the twelve – have an integral *Flo-2-C*. Of the two European lines lacking a functional *Flo-2-C*, one possesses an ORF destroyed by an insertion and in the second there is evidence for a recombination event that created a premature termination codon (lines 11, 13). Likewise *D. sechellia* lacks an ORF for transcript *Flo-2-C* due to a premature termination codon and to the same insertion found in the European *D. melanogaster* sample.

## Discussion

### Sequence Analysis Across the *Flo-2* Region

Ometto *et al*. [14] analyzed X-linked chromosome-wide patterns of DNA variation in the European population of *D. melanogaster* and found candidate loci that deviated from the demographic null model. In this follow-up study, we used one of these candidates to characterize the detailed pattern of nucleotide diversity and detect the possible action of positive selection.

Nucleotide diversity of the fully sequenced *Flo-2* region in the European sample shows a selective sweep-like pattern: a valley of reduced genetic variation of around 10 kb in size and flanking parts that steadily increase to neutral levels of European variation. Tajima’s *D* statistic is strongly negative for the European population and, in some segments, significantly different from zero. The *Flo-2* region is characterized by a high density of genes that are functionally and structurally well-conserved, suggesting strong evolutionary constraints. Divergence to *D. sechellia* remains constant at around 6% and thus excludes purifying selection and/or low mutation rates as major causes of low genetic diversity. Furthermore, the corresponding region in the African sample agrees well with the average value for X-linked nucleotide diversity without any appreciable decline. Thus it is unlikely that the low genetic variability of the European population is a consequence of an ancestral low polymorphism.

### Selective Sweep Analysis

We used two statistical tests for the sweep analysis of the *Flo-2* region: ω and SweepFinder. Both statistics capture different aspects of the data: ω is based on LD, while the CLR of SweepFinder is dependent on the SFS. Statistical significance was inferred using neutral coalescent simulations that were based on a slightly modified version of the demographic model of Laurent *et al.* [30]. SweepFinder revealed a statistically significant departure from neutral expectations of the European sample, suggesting that the *Flo-2* region has been a target of positive selection during the recent history of the European population. The results of the LD analysis were only marginally significant. In the following we discuss the genetic processes that have occurred in this part of the *Flo-2* region and may have created novelties on which selection has possibly operated.

### European Genetic Novelties

The European population sample has eight fixed nucleotide substitutions, all being private and derived, and the majority of which (six out of eight) are located in intronic regions of the *Flo-2* gene. The results of previous studies indicate that nucleotide and indel changes in introns can affect gene expression [44], [45], [46]. Interestingly, the second intron of *Flo-2* harbors the genes of the GMC oxidoredutase cluster (including *Eo* and *CG9503*), suggesting that they may indeed contain functional regulatory elements. Gene expression analysis of our European and African population samples revealed, however, no significant differences in the levels of *Eo* expression between populations for males [47], and only marginally for females [48]. Nonetheless, both *Eo* and *CG9503* are highly expressed only during embryonic and metamorphic development and have tissue-specific expression [18]. Similarly, *Flo-2* experiences a restricted tissue-specific expression in later developmental stages. As *CG9503*, *Flo-2* is in fact expressed in the wing disc, where its over-expression leads to detrimental effects during the development of wings [26]. A closer examination of intergenic and intronic regions around the GMC oxidoreductase cluster and the study of the development- and tissue-specific expression patterns may ultimately reveal the existence of regulatory elements and their role in the adaptive history of *Drosophila*.

In *D. melanogaster* the *Flo-2* gene has experienced a large transcript diversification, with isoforms that have distinct expression patterns. While the transcripts *Flo-2-A/E* and *Flo-2-B/F* are continuously expressed in larvae and adult flies, expression of *Flo-2-C* was shown to be restricted to larvae and pupae [49]. Interestingly, our results revealed that a functional *Flo-2-C* is shared by fewer African lines (three out of twelve) than European lines (ten out of twelve), although it is premature to associate this observation to adaptive processes. Since *Flo-2-C* is just one of the several functional isoforms of *Flo-2*, it does not suffer the genetic load typical of nonsense mRNA. Under such relaxed conditions negative selection might be negligible and facilitates the diversifications of the *Flo-2* gene. For instance, another premature termination codon was found in the *Flo-2-G* transcript of *D. melanogaster* (Figure 1, gene map) with a surprisingly high population frequency of 31.6%. Neumann-Giesen *et al.* [25] showed that a truncated Flotillin-2 has the ability to form homo-oligomers that enhance membrane association with full-length Flotillin-2. Thus, *Flo-2-C* might be a new gene variant whose gene product extends the function of the original *Flo-2*.

## Supporting Information

Table S1 **Set of primers, which were used for DNA amplification and sequencing of the** ***Flo-2*** **region.** Primer design was based on the *D. melanogaster* genome (FlyBase, Release 5.1).
(DOC)

Table S2 **Prior distribution of the parameters of the neutral demographic model inferred by ABC estimation for X-linked and autosomal data.**
(DOC)

Table S3 **DNA sequence alignment of the upper part of the** ***Flo-2-C*** **transcript (+46) in** ***D. melanogaster*** **and** ***D. sechellia*** **(** ***D. sec*** **).** African lines (A-number) show high levels of nonsense mutations (▪) due to frame shifts (X) or premature stop codons (*) compared to the European lines (E-number) which mostly show intact transcripts (▸). Codons (shaded in grey) specify an amino acid (below each codon, single letter code, not shaded).
(DOC)
