# Supplementary material for: Selective Sweep in the Flotillin-2 Region of European Drosophila melanogaster
Source: PLoS One. 2013 Feb 21;8(2):e56629. doi: 10.1371/journal.pone.0056629 (PMC3578937; doi:10.1371/journal.pone.0056629)
Supplement: Table S1 — Set of primers, which were used for DNA amplification and sequencing of the Flo-2 region. Primer design was based on the D. melanogaster genome (FlyBase, Release 5.1). (DOC) [file pone.0056629.s001.doc]

| ID | Forward Primer | Reverse Primer | Annealing  temperature |
| --- | --- | --- | --- |
| X-01228 | CGCAAATATTGAATACCTCTT | CCGTGGGCACTTGTCGCATGG | 50.9 |
| X-01229 | CCTCCCGATCGTCGAGAATCA | TGGCGAGGCTGTAGAGGATCA | 58.5 |
| X-01230 | GCTTCCAAAGTCGTAGACCAC | ATTGTGCCGTCAACAGTACAC | 55.4 |
| X-01231 | CTGAGCCTGAATCAACTCTGG | CTCTCGCCTGCAACAACGTCT | 56.6 |
| X-01232 | CAGCCGCATTTAGTAGTACCC | CCTGGCCGCTGCAACACGAAC | 57 |
| X-01233 | ACAGCTCACTCAATCGACTGG | ATCGCCTAAATGTCACTTATC | 52.5 |
| X-01234 | AGCGTCATGAAATGATCTTAC | GGATGCCAAGCTAAGGGTCTA | 50.6 |
| X-01235 | CACAGGCAATCATACTCAGTT | GATCGAGGGCGTGAAGATAGC | 53.8 |
| X-01236 | ACCGGGTGATAGATGGTAGAC | TTGGGTGGCGTGGAGGGATTG | 55.6 |
| X-01237 | AGCATCAGTTAATCCATGAGC | TCGGAGTGGAGTTCGTTAAGG | 55 |
| X-01238 | CGCCCAGACCAATGTGATCCT | GGCCAATACACCATATCATGC | 56.5 |
| X-01239 | TATGCAAATTTGGACGTAATC | GACGGAACCATCGGGAACATC | 54.1 |
| X-01240 | ATAGCCTCCCAGTTGTCGTAG | ATCCTCGACCATTATGACTTT | 54.3 |
| X-01241 | ACGATCCCTTAGGCAGACGTA | CTGTTTGCATTACCCATTAGC | 55.1 |
| X-01242 | CCGCTTCCCATTTAGTTAGGG | TGACGAAGCGTATCACGATAG | 52.2 |
| X-01243 | GTGATCGATAAATCTAAGTGG | ACTGGTTGCAAGTGAGAGGAC | 50.8 |
| X-01244 | TGATGGTGCATCTTTGGATAG | AAACGCAACATCAGTAACAGG | 51.3 |
| X-01245 | GTTTGCCTAGGTACAGTGGTA | CGGCAATTCTTCGACGCTTAC | 49.6 |
| X-01406 | TCGGAACATATGTAATGCCTATT | CCGCCAAGGGAATTGTGACGAAA | 52.0 |
| X-01407 | CCAAAGTGAAATGCGTAACAGTA | TCGATGGAGAGAGGAGCGGTGAA | 54.0 |
| X-01408 | GCGGCATGAATCACATTCGTTGA | ACCCAACTTTTCCTTTACCTACC | 54.6 |
| X-01409 | TGGCACACTTTATTTCGCTCTCT | TTGGGTGACGTCACACGCACAGT | 55.8 |
| X-01247 | CTCTCCATCGAACTCTCATAC | CACATCGGATTCAGGGTCATC | 52.1 |
| X-01248 | AAAGCAAAGCAAGTTACTGAC | CGGGTGCAGGCAGGATACCAA | 52.9 |
| X-01249 | TCTCCGCTCGTTTGTGGTTAT | TGTTCGATGAGCTAGTTCTAC | 51.9 |
| X-01250 | GAGTGCCGAAAGTAGCGAGTT | GAATCAATTGATGTGAGTGAA | 51.2 |
| X-01251 | CTGCGACTGAAGACCGACTTT | TGTGTGGGTGGTTAGGTTAGG | 51 |
| X-01252 | AATCGCTGCCCAAGGTTAGTG | CTAGGACACCACACAGACGAG | 53.3 |
| X-01333 | CTGCCCAAGGTTAGTGCTCGATATG | GCACCTTATATGGCACAATTCCGTA | 54.1 |
| X-01254 | AACCATTTAAGCAGAGTGTTA | TAAATGCATATTCGTAGTTCA | 47.6 |
| X-01255 | CAATTGGCGCTGCTTTACACC | TTGCTTTCTCCATCAGACTGC | 50 |
| X-01256 | ACAACACATATTGAACTACGA | TGCGTGCACTCTAACCTTTGA | 47.5 |
| X-01257 | AACTGGTTCATTTCGTCTTTA | TCAATCAGGAATGGTACACAG | 50.9 |
| X-01258 | GTGCACGCATAAAGATTACAC | AAGGAGCTGATTAAGGTGAAG | 54.5 |
| X-01259 | CCATTCCTGATTGAGACGAAC | TATGGCATGACAGAGGCTTCT | 58.4 |
| X-01260 | AGACCGTCCTCGTCGTAGAAG | CGGAAACCTATATTCGATAGC | 54.2 |
