# Supplementary material for: Selective Sweep in the Flotillin-2 Region of European Drosophila melanogaster
Source: PLoS One. 2013 Feb 21;8(2):e56629. doi: 10.1371/journal.pone.0056629 (PMC3578937; doi:10.1371/journal.pone.0056629)
Supplement: Table S2 — Prior distribution of the parameters of the neutral demographic model inferred by ABC estimation for X-linked and autosomal data. (DOC) [file pone.0056629.s002.doc]

| **Parameter** | **Prior Distribution** | | |
| --- | --- | --- | --- |
|  | **Min** | **Max** | **Distribution** |
| **Sizes** |  |  |  |
| Current African size | 10^5^ | 3 * 10^7^ | uniform |
| Current European size | 10^4^ | 5 * 10^6^ | uniform |
| Current Asian size | 10^4^ | 5 * 10^6^ | uniform |
| Bottleneck size of the Asian population | 10 | 10^5^ | uniform |
| Bottleneck size of the European population | 10 | 10^5^ | uniform |
| Size of the African ancestral population | 10^5^ | 2 * 10^7^ | uniform |
| **Times** |  |  |  |
| Exit out of Africa | 10^3^ | 10^6^ | uniform |
| Divergence between European and Asian populations | 10^3^ | Exit out of Africa | uniform |
| Expansion time of the African population | 10^2^ | 4 * 10^5^ | uniform |
