# Supplementary material for: Selective Sweep in the Flotillin-2 Region of European Drosophila melanogaster
Source: PLoS One. 2013 Feb 21;8(2):e56629. doi: 10.1371/journal.pone.0056629 (PMC3578937; doi:10.1371/journal.pone.0056629)
Supplement: Table S3 — DNA sequence alignment of the upper part of the Flo-2-C transcript (+46) in D. melanogaster and D. sechellia ( D. sec ). African lines (A-number) show high levels of nonsense mutations (▪) due to frame shifts (X) or premature stop codons (*) compared to the European lines (E-number) which mostly show intact transcripts (▸). Codons (shaded in grey) specify an amino acid (below each codon, single letter code, not shaded). (DOC) [file pone.0056629.s003.doc]

| Rel codon pos 12950 | | | | | 12953 | | | 12956 | | | 12959 | | | 12962 | | | 12965 | | | 12968 | | | 12971 | | | 12974 | | |
| --- | --- | --- | --- | --- | --- | --- | --- | --- | --- | --- | --- | --- | --- | --- | --- | --- | --- | --- | --- | --- | --- | --- | --- | --- | --- | --- | --- | --- |
| *D. sec* |  | G | A | A | - | - | - | - | - | - | - | - | - | - | - | - | C | C | C | A | C | A | C | A | C | A | C | A |
|  | ■ | E |  |  |  |  |  |  |  |  |  |  |  |  |  |  | P |  |  | T |  |  | H |  |  | T |  |  |
| A082 |  | G | A | A | A | C | C | - | - | - | - | - | - | A | C | A | C | A | C | A | C | A | C | A | C | A | C | A |
|  | ■ | E |  |  | T |  |  |  |  |  |  |  |  | T |  |  | H |  |  | T |  |  | H |  |  | T |  |  |
| A084 |  | G | A | A | A | C | C | - | - | - | - | - | - | - | - | - | - | - | - | A | C | A | C | A | C | A | C | A |
|  | ■ | E |  |  | T |  |  |  |  |  |  |  |  |  |  |  |  |  |  | T |  |  | H |  |  | T |  |  |
| A095 |  | G | A | A | A | C | C | A | C | A | C | A | C | A | C | A | C | A | C | A | C | A | C | A | C | A | C | A |
|  | ■ | E |  |  | T |  |  | T |  |  | H |  |  | T |  |  | H |  |  | T |  |  | H |  |  | T |  |  |
| A131 |  | G | A | A | A | C | C | A | C | A | C | A | C | A | C | A | C | A | C | A | C | A | C | A | C | A | C | A |
|  | ■ | E |  |  | T |  |  | T |  |  | H |  |  | T |  |  | H |  |  | T |  |  | H |  |  | T |  |  |
| A145 |  | G | A | A | A | C | C | - | - | - | - | - | - | - | - | - | - | - | - | A | C | A | C | A | C | A | C | A |
|  | ■ | E |  |  | T |  |  |  |  |  |  |  |  |  |  |  |  |  |  | T |  |  | H |  |  | T |  |  |
| A157 |  | G | A | A | A | C | C | A | C | A | C | A | C | A | C | A | C | A | C | A | C | A | C | A | C | A | C | A |
|  | ■ | E |  |  | T |  |  | T |  |  | H |  |  | T |  |  | H |  |  | T |  |  | H |  |  | T |  |  |
| A186 |  | G | A | A | A | C | C | A | C | A | C | A | C | A | C | A | C | A | C | A | C | A | C | A | C | A | C | A |
|  | ► | E |  |  | T |  |  | T |  |  | H |  |  | T |  |  | H |  |  | T |  |  | H |  |  | T |  |  |
| A191 |  | G | A | A | A | C | C | A | C | A | C | A | C | A | C | A | C | A | C | A | C | A | C | A | C | A | C | A |
|  | ■ | E |  |  | T |  |  | T |  |  | H |  |  | T |  |  | H |  |  | T |  |  | H |  |  | T |  |  |
| A229 |  | G | A | A | A | C | C | A | C | A | C | A | C | A | C | A | C | A | C | A | C | A | C | A | C | A | C | A |
|  | ■ | E |  |  | T |  |  | T |  |  | H |  |  | T |  |  | H |  |  | T |  |  | H |  |  | T |  |  |
| A377 |  | G | A | A | A | C | C | A | C | A | C | A | C | A | C | A | C | A | C | A | C | A | C | A | C | A | C | A |
|  | ► | E |  |  | T |  |  | T |  |  | H |  |  | T |  |  | H |  |  | T |  |  | H |  |  | T |  |  |
| A384 |  | G | A | A | A | C | C | A | C | A | C | A | C | A | C | A | C | A | C | A | C | A | C | A | C | A | C | A |
|  | ► | E |  |  | T |  |  | T |  |  | H |  |  | T |  |  | H |  |  | T |  |  | H |  |  | T |  |  |
| A398 |  | G | A | A | A | C | C | A | C | A | C | A | C | A | C | A | C | A | C | A | C | A | C | A | C | A | C | A |
|  | ■ | E |  |  | T |  |  | T |  |  | H |  |  | T |  |  | H |  |  | T |  |  | H |  |  | T |  |  |
| E01 |  | G | A | A | A | C | C | A | C | A | C | A | C | A | C | A | C | A | C | A | C | A | C | A | C | A | C | A |
|  | ► | E |  |  | T |  |  | T |  |  | H |  |  | T |  |  | H |  |  | T |  |  | H |  |  | T |  |  |
| E02 |  | G | A | A | A | C | C | A | C | A | C | A | C | A | C | A | C | A | C | A | C | A | C | A | C | A | C | A |
|  | ► | E |  |  | T |  |  | T |  |  | H |  |  | T |  |  | H |  |  | T |  |  | H |  |  | T |  |  |
| E11 |  | G | A | A | A | C | C | A | C | A | C | A | C | A | C | A | C | A | C | A | C | A | C | A | C | A | C | A |
|  | ■ | E |  |  | T |  |  | T |  |  | H |  |  | T |  |  | H |  |  | T |  |  | H |  |  | T |  |  |
| E12 |  | G | A | A | A | C | C | A | C | A | C | A | C | A | C | A | C | A | C | A | C | A | C | A | C | A | C | A |
|  | ► | E |  |  | T |  |  | T |  |  | H |  |  | T |  |  | H |  |  | T |  |  | H |  |  | T |  |  |
| E13 |  | G | A | A | A | C | C | A | C | A | C | A | C | A | C | A | C | A | C | A | C | A | C | A | C | A | C | A |
|  | ■ | E |  |  | T |  |  | T |  |  | H |  |  | T |  |  | H |  |  | T |  |  | H |  |  | T |  |  |
| E14 |  | G | A | A | A | C | C | A | C | A | C | A | C | A | C | A | C | A | C | A | C | A | C | A | C | A | C | A |
|  | ► | E |  |  | T |  |  | T |  |  | H |  |  | T |  |  | H |  |  | T |  |  | H |  |  | T |  |  |
| E15 |  | G | A | A | A | C | C | A | C | A | C | A | C | A | C | A | C | A | C | A | C | A | C | A | C | A | C | A |
|  | ► | E |  |  | T |  |  | T |  |  | H |  |  | T |  |  | H |  |  | T |  |  | H |  |  | T |  |  |
| E16 |  | G | A | A | A | C | C | A | C | A | C | A | C | A | C | A | C | A | C | A | C | A | C | A | C | A | C | A |
|  | ► | E |  |  | T |  |  | T |  |  | H |  |  | T |  |  | H |  |  | T |  |  | H |  |  | T |  |  |
| E17 |  | G | A | A | A | C | C | A | C | A | C | A | C | A | C | A | C | A | C | A | C | A | C | A | C | A | C | A |
|  | ► | E |  |  | T |  |  | T |  |  | H |  |  | T |  |  | H |  |  | T |  |  | H |  |  | T |  |  |
| E18 |  | G | A | A | A | C | C | A | C | A | C | A | C | A | C | A | C | A | C | A | C | A | C | A | C | A | C | A |
|  | ► | E |  |  | T |  |  | T |  |  | H |  |  | T |  |  | H |  |  | T |  |  | H |  |  | T |  |  |
| E19 |  | G | A | A | A | C | C | A | C | A | C | A | C | A | C | A | C | A | C | A | C | A | C | A | C | A | C | A |
|  | ► | E |  |  | T |  |  | T |  |  | H |  |  | T |  |  | H |  |  | T |  |  | H |  |  | T |  |  |
| E20 |  | G | A | A | A | C | C | A | C | A | C | A | C | A | C | A | C | A | C | A | C | A | C | A | C | A | C | A |
|  | ► | E |  |  | T |  |  | T |  |  | H |  |  | T |  |  | H |  |  | T |  |  | H |  |  | T |  |  |

| 12977 | | | 12980 | | | 12983 | | | 12986 | | | 12989 | | | 12992 | | | 12995 | | | 12998 | | | 13001 | | | 13004 | | |
| --- | --- | --- | --- | --- | --- | --- | --- | --- | --- | --- | --- | --- | --- | --- | --- | --- | --- | --- | --- | --- | --- | --- | --- | --- | --- | --- | --- | --- | --- |
| A | A | C | A | A | A | - | - | - | - | - | - | - | - | - | - | - | - | - | - | - | - | - | - | - | - | - | - | - | - |
| N |  |  | K |  |  |  |  |  |  |  |  |  |  |  |  |  |  |  |  |  |  |  |  |  |  |  |  |  |  |
| C | A | C | A | A | A | C | A | C | T | - | - | - | - | - | - | - | - | - | - | - | - | - | - | - | - | - | - | - | - |
| H |  |  | K |  |  | H |  |  | S |  |  |  |  |  |  |  |  |  |  |  |  |  |  |  |  |  |  |  |  |
| C | A | C | A | A | A | C | A | C | T | - | - | - | - | - | - | - | - | - | - | - | - | - | - | - | - | - | - | - | - |
| H |  |  | K |  |  | H |  |  | S |  |  |  |  |  |  |  |  |  |  |  |  |  |  |  |  |  |  |  |  |
| C | A | C | A | A | A | C | A | C | T | - | - | - | - | - | - | - | - | - | - | - | - | - | - | - | - | - | - | - | - |
| H |  |  | K |  |  | H |  |  | S |  |  |  |  |  |  |  |  |  |  |  |  |  |  |  |  |  |  |  |  |
| C | - | - | A | A | A | C | A | C | T | - | - | - | - | - | - | - | - | - | - | - | - | - | - | - | - | - | - | - | - |
| Q |  |  |  |  | T |  |  | L |  |  |  |  |  |  |  |  |  |  |  |  |  |  |  |  |  |  |  |  |  |
| C | A | C | A | A | A | C | A | C | T | - | - | - | - | - | - | - | - | - | - | - | - | - | - | - | - | - | - | - | - |
| H |  |  | K |  |  | H |  |  | S |  |  |  |  |  |  |  |  |  |  |  |  |  |  |  |  |  |  |  |  |
| C | A | C | A | A | A | C | A | C | T | - | - | - | - | - | - | - | - | - | - | - | - | - | - | - | - | - | - | - | - |
| H |  |  | K |  |  | H |  |  | S |  |  |  |  |  |  |  |  |  |  |  |  |  |  |  |  |  |  |  |  |
| C | A | C | A | A | A | C | - | - | - | - | - | - | - | - | - | - | - | - | - | - | - | - | - | - | - | - | - | - | - |
| H |  |  | K |  |  | H |  |  |  |  |  |  |  |  |  |  |  |  |  |  |  |  |  |  |  |  |  |  |  |
| C | A | C | A | A | A | C | A | C | T | - | - | - | - | - | - | - | - | - | - | - | - | - | - | - | - | - | - | - | - |
| H |  |  | K |  |  | H |  |  | S |  |  |  |  |  |  |  |  |  |  |  |  |  |  |  |  |  |  |  |  |
| C | A | C | A | A | A | C | A | C | T | - | - | - | - | - | - | - | - | - | - | - | - | - | - | - | - | - | - | - | - |
| H |  |  | K |  |  | H |  |  | S |  |  |  |  |  |  |  |  |  |  |  |  |  |  |  |  |  |  |  |  |
| C | A | C | A | A | A | C | A | C | T | - | - | - | - | - | - | - | - | - | - | - | - | - | - | - | - | - | - | - | - |
| H |  |  | K |  |  | H |  |  | S |  |  |  |  |  |  |  |  |  |  |  |  |  |  |  |  |  |  |  |  |
| C | A | C | A | A | A | C | A | C | T | - | - | - | - | - | - | - | - | - | - | - | - | - | - | - | - | - | - | - | - |
| H |  |  | K |  |  | H |  |  | S |  |  |  |  |  |  |  |  |  |  |  |  |  |  |  |  |  |  |  |  |
| C | A | C | A | A | A | C | A | C | T | - | - | - | - | - | - | - | - | - | - | - | - | - | - | - | - | - | - | - | - |
| H |  |  | K |  |  | H |  |  | S |  |  |  |  |  |  |  |  |  |  |  |  |  |  |  |  |  |  |  |  |
| C | - | - | A | A | A | C | A | C | T | - | - | - | - | - | - | C | A | C | A | C | A | C | A | C | A | C | A | C | A |
| Q |  |  |  |  | T |  |  | L |  |  |  |  |  |  |  |  | T |  |  | H |  |  | T |  |  | H |  |  | T |
| C | - | - | A | A | A | C | A | C | T | - | - | - | - | - | - | C | A | C | A | C | A | C | A | C | A | C | A | C | A |
| Q |  |  |  |  | T |  |  | L |  |  |  |  |  |  |  |  | T |  |  | H |  |  | T |  |  | H |  |  | T |
| C | A | C | A | A | A | C | A | C | T | C | A | C | A | C | A | C | A | C | A | C | A | C | A | C | A | C | A | C | A |
| H |  |  | K |  |  | H |  |  | S |  |  | H |  |  | T |  |  | H |  |  | T |  |  | H |  |  | T |  |  |
| C | - | - | A | A | A | C | A | C | T | - | - | - | - | - | - | C | A | C | A | C | A | C | A | C | A | C | A | C | A |
| Q |  |  |  |  | T |  |  | L |  |  |  |  |  |  |  |  | T |  |  | H |  |  | T |  |  | H |  |  | T |
| C | A | C | A | A | A | C | A | C | T | - | - | - | - | - | - | C | A | C | A | C | A | C | A | C | A | C | A | C | A |
| H |  |  | K |  |  | H |  |  | S |  |  |  |  |  |  |  |  | H |  |  | T |  |  | H |  |  | T |  |  |
| C | - | - | A | A | A | C | A | C | T | - | - | - | - | - | - | C | A | C | A | C | A | C | A | C | A | C | A | C | A |
| Q |  |  |  |  | T |  |  | L |  |  |  |  |  |  |  |  | T |  |  | H |  |  | T |  |  | H |  |  | T |
| C | - | - | A | A | A | C | A | C | T | - | - | - | - | - | - | C | A | C | A | C | A | C | A | C | A | C | A | C | A |
| Q |  |  |  |  | T |  |  | L |  |  |  |  |  |  |  |  | T |  |  | H |  |  | T |  |  | H |  |  | T |
| C | - | - | A | A | A | C | A | C | T | - | - | - | - | - | - | C | A | C | A | C | A | C | A | C | A | C | A | C | A |
| Q |  |  |  |  | T |  |  | L |  |  |  |  |  |  |  |  | T |  |  | H |  |  | T |  |  | H |  |  | T |
| C | - | - | A | A | A | C | A | C | T | - | - | - | - | - | - | C | A | C | A | C | A | C | A | C | A | C | A | C | A |
| Q |  |  |  |  | T |  |  | L |  |  |  |  |  |  |  |  | T |  |  | H |  |  | T |  |  | H |  |  | T |
| C | - | - | A | A | A | C | A | C | T | - | - | - | - | - | - | C | A | C | A | C | A | C | A | C | A | C | A | C | A |
| Q |  |  |  |  | T |  |  | L |  |  |  |  |  |  |  |  | T |  |  | H |  |  | T |  |  | H |  |  | T |
| C | - | - | A | A | A | C | A | C | T | - | - | - | - | - | - | C | A | C | A | C | A | C | A | C | A | C | A | C | A |
| Q |  |  |  |  | T |  |  | L |  |  |  |  |  |  |  |  | T |  |  | H |  |  | T |  |  | H |  |  | T |
| C | - | - | A | A | A | C | A | C | T | - | - | - | - | - | - | C | A | C | A | C | A | C | A | C | A | C | A | C | A |
| Q |  |  |  |  | T |  |  | L |  |  |  |  |  |  |  |  | T |  |  | H |  |  | T |  |  | H |  |  | T |
|  |  |  |  |  |  |  |  |  |  |  |  | Insertion: | | | | 1 | 2 | 3 | 4 | 5 | 6 | 7 | 8 | 9 | 10 | 11 | 12 | 13 | 14 |

|  | 13007 | | | 13010 | | | 13013 | | | 13016 | | | 13019 | | | 13022 | | | 13025 | | | 13028 | | | 13031 | | |
| --- | --- | --- | --- | --- | --- | --- | --- | --- | --- | --- | --- | --- | --- | --- | --- | --- | --- | --- | --- | --- | --- | --- | --- | --- | --- | --- | --- |
| D. sec | - | - | - | - | - | - | - | - | C | T | C | A | C | A | C | A | C | A | C | A | C | A | C | - | - | - | - |
|  |  |  |  |  |  |  |  |  | L |  |  | T |  |  | H |  |  | T |  |  | H |  |  |  |  |  |  |
| A082 | - | - | - | - | - | - | - | - | C | T | C | A | C | A | C | A | C | A | A | A | C | A | - | - | - | - | - |
|  |  |  |  |  |  |  |  |  |  |  | H |  |  | T |  |  | Q |  |  | T |  |  |  |  |  |  |  |
| A084 | - | - | - | - | - | - | - | - | C | T | C | A | C | A | C | A | C | A | C | A | C | A | T | A | A | A | C |
|  |  |  |  |  |  |  |  |  |  |  | H |  |  | T |  |  | H |  |  | T |  |  | * |  |  | T |  |
| A095 | - | - | - | - | - | - | - | - | - | - | - | - | C | A | C | A | C | A | C | A | C | A | C | A | A | A | C |
|  |  |  |  |  |  |  |  |  |  |  |  |  |  |  | H |  |  | T |  |  | H |  |  | K |  |  | H |
| A131 | - | - | - | - | - | - | C | T | C | A | C | A | C | A | C | A | C | A | C | A | C | A | T | A | A | A | C |
|  |  |  |  |  |  |  |  | S |  |  | H |  |  | T |  |  | H |  |  | T |  |  | * |  |  | T |  |
| A145 | - | - | - | - | - | - | - | - | C | T | C | A | C | A | C | A | C | A | C | A | C | A | T | A | A | A | C |
|  |  |  |  |  |  |  |  |  |  |  | H |  |  | T |  |  | H |  |  | T |  |  | * |  |  | T |  |
| A157 | - | - | - | - | - | - | - | - | - | - | - | - | C | A | C | A | C | A | C | A | C | A | C | A | A | A | C |
|  |  |  |  |  |  |  |  |  |  |  |  |  |  |  | H |  |  | T |  |  | H |  |  | K |  |  | H |
| A186 | - | - | - | - | - | - | - | - | - | - | - | - | - | - | - | - | - | - | - | - | - | - | - | - | - | - | - |
|  |  |  |  |  |  |  |  |  |  |  |  |  |  |  |  |  |  |  |  |  |  |  |  |  |  |  |  |
| A191 | - | - | - | - | - | - | - | - | C | T | C | A | C | A | C | A | C | A | C | A | C | A | T | A | A | A | C |
|  |  |  |  |  |  |  |  |  |  |  | H |  |  | T |  |  | H |  |  | T |  |  | * |  |  | T |  |
| A229 | - | - | - | - | - | - | - | - | C | T | C | A | C | A | C | A | C | A | C | A | C | A | T | A | A | A | C |
|  |  |  |  |  |  |  |  |  |  |  | H |  |  | T |  |  | H |  |  | T |  |  | * |  |  | T |  |
| A377 | - | - | - | - | - | - | C | T | C | A | C | A | C | A | C | A | C | A | C | A | C | A | T | A | A | A | C |
|  |  |  |  |  |  |  |  |  | H |  |  | T |  |  | H |  |  | T |  |  | H |  |  | K |  |  | H |
| A384 | - | - | - | - | - | - | C | T | C | A | C | A | C | A | C | A | C | A | C | A | C | A | T | A | A | A | C |
|  |  |  |  |  |  |  |  |  | H |  |  | T |  |  | H |  |  | T |  |  | H |  |  | K |  |  | H |
| A398 | - | - | - | - | - | - | - | - | C | T | C | A | C | A | C | A | C | A | C | A | C | A | T | A | A | A | C |
|  |  |  |  |  |  |  |  |  |  |  | H |  |  | T |  |  | H |  |  | T |  |  | * |  |  | T |  |
| E01 | C | A | A | A | C | A | C | T | C | T | C | A | C | A | C | A | C | A | C | A | C | A | T | A | A | A | C |
|  |  |  | N |  |  | T |  |  | L |  |  | T |  |  | H |  |  | T |  |  | H |  |  | K |  |  | H |
| E02 | C | A | A | A | C | A | C | T | C | T | C | A | C | A | C | A | C | A | C | A | C | A | T | A | A | A | C |
|  |  |  | N |  |  | T |  |  | L |  |  | T |  |  | H |  |  | T |  |  | H |  |  | K |  |  | H |
| E11 | C | A | A | A | C | A | C | T | C | T | C | A | C | A | C | A | C | A | C | A | C | A | T | A | A | A | C |
|  | Q |  |  | T |  |  | L |  |  | S |  |  | H |  |  | T |  |  | H |  |  | I |  |  | N |  |  |
| E12 | C | A | A | A | C | A | C | T | C | T | C | A | C | A | C | A | C | A | C | A | C | A | T | A | A | A | C |
|  |  |  | N |  |  | T |  |  | L |  |  | T |  |  | H |  |  | T |  |  | H |  |  | K |  |  | H |
| E13 | C | A | A | A | C | A | C | T | C | T | C | A | C | A | C | A | C | A | C | A | C | A | T | A | A | A | C |
|  | Q |  |  | T |  |  | L |  |  | S |  |  | H |  |  | T |  |  | H |  |  | I |  |  | N |  |  |
| E14 | C | A | A | A | C | A | C | T | C | T | C | A | C | A | C | A | C | A | C | A | C | A | T | A | A | A | C |
|  |  |  | N |  |  | T |  |  | L |  |  | T |  |  | H |  |  | T |  |  | H |  |  | K |  |  | H |
| E15 | C | A | A | A | C | A | C | T | C | T | C | A | C | A | C | A | C | A | C | A | C | A | T | A | A | A | C |
|  |  |  | N |  |  | T |  |  | L |  |  | T |  |  | H |  |  | T |  |  | H |  |  | K |  |  | H |
| E16 | C | A | A | A | C | A | C | T | C | T | C | A | C | A | C | A | C | A | C | A | C | A | T | A | A | A | C |
|  |  |  | N |  |  | T |  |  | L |  |  | T |  |  | H |  |  | T |  |  | H |  |  | K |  |  | H |
| E17 | C | A | A | A | C | A | C | T | C | T | C | A | C | A | C | A | C | A | C | A | C | A | T | A | A | A | C |
|  |  |  | N |  |  | T |  |  | L |  |  | T |  |  | H |  |  | T |  |  | H |  |  | K |  |  | H |
| E18 | C | A | A | A | C | A | C | T | C | T | C | A | C | A | C | A | C | A | C | A | C | A | T | A | A | A | C |
|  |  |  | N |  |  | T |  |  | L |  |  | T |  |  | H |  |  | T |  |  | H |  |  | K |  |  | H |
| E19 | C | A | A | A | C | A | C | T | C | T | C | A | C | A | C | A | C | A | C | A | C | A | T | A | A | A | C |
|  |  |  | N |  |  | T |  |  | L |  |  | T |  |  | H |  |  | T |  |  | H |  |  | K |  |  | H |
| E20 | C | A | A | A | C | A | C | T | C | T | C | A | C | A | C | A | C | A | C | A | C | A | T | A | A | A | C |
|  |  |  | N |  |  | T |  |  | L |  |  | T |  |  | H |  |  | T |  |  | H |  |  | K |  |  | H |
|  | 15 | 16 | 17 | 18 | 19 | 20 |  |  |  |  |  |  |  |  |  |  |  |  |  |  |  |  |  |  |  |  |  |

| 13034 | | | 13037 | | | 13040 | | | 13043 | | | 13046 | | | 13049 | | |  |  |  |
| --- | --- | --- | --- | --- | --- | --- | --- | --- | --- | --- | --- | --- | --- | --- | --- | --- | --- | --- | --- | --- |
| - | - | - | - | - | - | A | C | G | T | A | C | A | G | A | T | G | A | T | G | D. sec |
|  |  |  |  |  |  | T |  |  | Y |  |  | R |  |  | * |  |  |  |  |  |
| - | - | - | - | - | - | A | C | G | T | A | C | A | G | A | T | G | A | T | G | A082 |
|  |  |  |  |  |  | T |  |  | Y |  |  | R |  |  | * |  |  |  |  |  |
| A | C | A | A | A | C | A | C | G | T | A | C | A | G | A | T | G | A | T | G | A084 |
|  | Q |  |  | T |  |  | R |  |  | T |  |  | D |  |  | D |  |  | X |  |
| A | C | A | C | - | - | A | C | G | T | A | C | A | G | A | T | G | A | T | G | A095 |
|  |  | T |  |  |  |  | R |  |  | T |  |  | D |  |  | D |  |  | X |  |
| A | C | A | A | A | C | A | C | G | T | A | C | A | G | A | T | G | A | T | G | A131 |
|  | Q |  |  | T |  |  | R |  |  | T |  |  | D |  |  | D |  |  | X |  |
| A | C | A | A | A | C | A | C | G | T | A | C | A | G | A | T | G | A | T | G | A145 |
|  | Q |  |  | T |  |  | R |  |  | T |  |  | D |  |  | D |  |  | X |  |
| A | C | A | C | - | - | A | C | G | T | A | C | A | G | A | T | G | A | T | G | A157 |
|  |  | T |  |  |  |  | R |  |  | T |  |  | D |  |  | D |  |  | X |  |
| - | - | - | - | - | - | A | C | G | T | A | C | A | G | A | T | G | A | T | G | A186 |
|  |  |  |  |  |  |  |  | V |  |  | Q |  |  | M |  |  | M |  |  |  |
| A | C | A | A | A | C | A | C | G | T | A | C | A | G | A | T | G | A | T | G | A191 |
|  | Q |  |  | T |  |  | R |  |  | T |  |  | D |  |  | D |  |  | X |  |
| A | C | A | A | A | C | A | C | G | T | A | C | A | G | A | T | G | A | T | G | A229 |
|  | Q |  |  | T |  |  | R |  |  | T |  |  | D |  |  | D |  |  | X |  |
| A | C | A | A | A | C | A | C | G | T | A | C | A | G | A | T | G | A | T | G | A377 |
|  |  | K |  |  | H |  |  | V |  |  | Q |  |  | M |  |  | M |  |  |  |
| A | C | A | A | A | C | A | C | G | T | A | C | A | G | A | T | G | A | T | G | A384 |
|  |  | K |  |  | H |  |  | V |  |  | Q |  |  | M |  |  | M |  |  |  |
| A | C | A | A | A | C | A | C | G | T | A | C | A | G | A | T | G | A | T | G | A398 |
|  | Q |  |  | T |  |  | R |  |  | T |  |  | D |  |  | D |  |  | X |  |
| A | C | A | A | A | C | A | C | G | T | A | C | A | G | A | T | G | A | T | G | E01 |
|  |  | K |  |  | H |  |  | V |  |  | Q |  |  | M |  |  | M |  |  |  |
| A | C | A | A | A | C | A | C | G | T | A | C | A | G | A | T | G | A | T | G | E02 |
|  |  | K |  |  | H |  |  | V |  |  | Q |  |  | M |  |  | M |  |  |  |
| A | C | A | A | A | C | A | C | G | T | A | C | A | G | A | T | G | A | T | G | E11 |
| T |  |  | N |  |  | T |  |  | Y |  |  | R |  |  | * |  |  |  |  |  |
| A | C | A | A | A | C | A | C | G | T | A | C | A | G | A | T | G | A | T | G | E12 |
|  |  | K |  |  | H |  |  | V |  |  | Q |  |  | M |  |  | M |  |  |  |
| A | C | A | A | A | C | A | C | G | T | A | C | A | G | A | T | G | A | T | G | E13 |
| T |  |  | N |  |  | T |  |  | Y |  |  | R |  |  | * |  |  |  |  |  |
| A | C | A | A | A | C | A | C | G | T | A | C | A | G | A | T | G | A | T | G | E14 |
|  |  | K |  |  | H |  |  | V |  |  | Q |  |  | M |  |  | M |  |  |  |
| A | C | A | A | A | C | A | C | G | T | A | C | A | G | A | T | G | A | T | G | E15 |
|  |  | K |  |  | H |  |  | V |  |  | Q |  |  | M |  |  | M |  |  |  |
| A | C | A | A | A | C | A | C | G | T | A | C | A | G | A | T | G | A | T | G | E16 |
|  |  | K |  |  | H |  |  | V |  |  | Q |  |  | M |  |  | M |  |  |  |
| A | C | A | A | A | C | A | C | G | T | A | C | A | G | A | T | G | A | T | G | E17 |
|  |  | K |  |  | H |  |  | V |  |  | Q |  |  | M |  |  | M |  |  |  |
| A | C | A | A | A | C | A | C | G | T | A | C | A | G | A | T | G | A | T | G | E18 |
|  |  | K |  |  | H |  |  | V |  |  | Q |  |  | M |  |  | M |  |  |  |
| A | C | A | A | A | C | A | C | G | T | A | C | A | G | A | T | G | A | T | G | E19 |
|  |  | K |  |  | H |  |  | V |  |  | Q |  |  | M |  |  | M |  |  |  |
| A | C | A | A | A | C | A | C | G | T | A | C | A | G | A | T | G | A | T | G | E20 |
|  |  | K |  |  | H |  |  | V |  |  | Q |  |  | M |  |  | M |  |  |  |
